# Neurodevelopmental and Psychiatric Disorders and the Use of Psychotropic Medications in a National Sample of Individuals With Juvenile Neuronal Ceroid Lipofuscinosis

**DOI:** 10.1177/08830738251413827

**Published:** 2026-02-05

**Authors:** Beate Oerbeck, Ingrid B. Helland, Heather R. Adams, Kristin Romvig Overgaard

**Affiliations:** 1 Division of Mental Health and Addiction, 155272 Oslo University Hospital, Oslo, Norway; 2 Division of Pediatric and Adolescent Medicine, 155272 Oslo University Hospital, Oslo, Norway; 3 Department of Neurology & Department of Pediatrics, 6923 University of Rochester Medical Center, Rochester, NY, USA; 4 Institute of Clinical Medicine, 6305 University of Oslo, Oslo, Norway

**Keywords:** Batten disease, psychiatric disorders, Spielmeyer-Vogt disease

## Abstract

Juvenile neuronal ceroid lipofuscinosis, linked to mutations in the ceroid lipofuscinosis, neuronal 3 (CLN3) gene, is a childhood-onset neurodegenerative disorder. Although mood and behavioral symptoms are described in this group, it's unclear whether these meet diagnostic criteria. We investigated the occurrence of neurodevelopmental and psychiatric disorders in a nationally representative sample, using a semistructured psychiatric interview, as well as the use of psychotropic medication. Ten of 20 individuals met the criteria for one or more current diagnoses, with an additional 5 having past diagnoses, resulting in a lifetime occurrence in 15 individuals. Anxiety disorders were the most frequent diagnostic group, followed by neurodevelopmental disorders. Attention-deficit hyperactivity disorder was the most common single diagnosis. Subthreshold psychiatric symptoms were present in all individuals. Although psychiatric disorders were frequent, few used psychotropic medication. These findings underscore the need for routine monitoring of neurodevelopmental and psychiatric disorders in individuals with CLN3 and the provision of evidence-based treatments.

The neuronal ceroid lipofuscinoses (NCLs) are a group of rare, recessively inherited lysosomal disorders characterized by overlapping clinical and pathologic features, including loss of vision, seizures, progressive motor and cognitive decline, and premature death typically occurring in the second or third decade of life.^1,2^ For the juvenile form, often referred to as Batten disease or ceroid lipofuscinosis, neuronal 3 (CLN3), symptom onset occurs around age 5-6 years.

The epidemiologic distribution of CLN3 disease is geographically related, with incidence rates across different countries ranging from 0.5 to 8.0 per 100 000 live births.^3^ In Norway, the prevalence has been estimated at 8.3 per million inhabitants, which is higher than that reported in Sweden, Denmark, Portugal, Italy, Germany, the United States, and Argentina, but lower than that in Finland and Iceland.^3^

It is well known that many children affected by CLN3 disease develop emotional and behavior problems such as anxiety, depression, aggression, and/or psychotic symptoms.^4–6^ Despite this, research on these symptoms remains limited. However, one study recently compared challenging behaviors in 2 of the most common subgroups of NCLs and confirmed that they were quite prevalent in CLN3 disease (65%) compared with the primarily late infantile form, CLN2 disease (25%).^7^ To date, only 2 studies on CLN3 disease (from the USA and Finland) have quantified and found elevated symptom levels using standardized questionnaires.^8,9^ The US study also identified obsessive-compulsive symptoms alongside routine-bound and ritualistic behaviors.^9^ In Norway, we published a case study of a child with CLN3 who benefited from treatment for diagnosed obsessive-compulsive disorder.^10^ Emotional problems and maladaptive and obsessive-compulsive behaviors were also found to be quite common in CLN3 using both the Vineland Adaptive Behavior Scales–3^7^ and in a qualitative interview with parents.^11^ However, no studies to date have employed a comprehensive parental psychiatric diagnostic interview to determine whether these and other psychiatric symptoms meet diagnostic criteria according to the *Diagnostic and Statistical Manual of Mental Disorders, Fifth Edition* (DSM-5). This approach is crucial for at least 2 reasons. First, relying on parental questionnaires to assess child psychiatric symptoms allows for subjective interpretation when parents are left on their own to interpret the questions. Instruments such as the Child Behavior Checklist, used in prior CLN3 studies,^12^ are intended for detecting symptoms rather than for diagnosing psychiatric disorders. In contrast, psychiatric interviews offer quantitative and qualitative insights to determine whether the reported symptoms align with the DSM-5 diagnostic criteria. Furthermore, interviewers can ask follow-up questions to clarify the respondent's meaning when needed. This is particularly important for individuals with CLN3 and other progressive neurologic disorders, where language, cognition, and somatic functions (eg, epilepsy, pain, and blindness) are impaired, further complicating interpretations of questionnaires. Second, interviews not only provide data on the occurrence of psychiatric disorders but may also be clinically useful by providing an overview of relevant questions to ask at medical checkups at different ages to aid in planning beneficial treatments and evaluating responses to treatment.

This project aimed to assess the occurrence of psychiatric disorders in a national sample of individuals with CLN3 and characterize the use of psychotropic medications. Based on the existing literature, we hypothesized that there would be a high occurrence rate of psychiatric disorders and that many individuals would be prescribed psychotropic medications to manage these disorders.

## Participants and Methods

### Participants

Participants eligible for inclusion were parents of all living individuals in Norway diagnosed with CLN3 through molecular genetic testing, excluding those diagnosed within the past year (see Ethics Approval). The parents provided information about the individuals with CLN3 and some sociodemographic data.

#### Procedures for recruitment

Parents of individuals with CLN3 were recruited from the Norwegian NCL user organization (https://www.nncl.no/) that comprise all but 1 known individuals with CLN3. Through the project's user representative, members were contacted and asked to participate; if interested, they were provided with a consent form to review. When the signed consent forms were returned to the investigators, background sociodemographic questionnaires were sent to the participating families and the time for a diagnostic telephone interview was planned.

### Measures

#### Background sociodemographic questionnaire

Parents provided information on the affected individual's sex, age at inclusion, and age of CLN3 symptom start. Additionally, they reported whether the offspring lived at home or in care, their own age and educational level and whether they worked full-time or not.

#### Information from medical records

All individuals with CLN3 are offered annual checkups at the Oslo University Hospital with the same child neurologist (author I.H.) who provided information from medical records for participants’ CLN3-symptom evaluation and present medication use (including indication).

#### CLN3 symptom evaluation

The Hamburg Kohlschütter scale was used to assess CLN3.^13^ The scale evaluates 5 core symptom domains: seizures, intellectual ability, language, motor function, and epilepsy (grand mal seizures only). Each domain is rated on a 4-point scale (0-3), with a higher score indicating better function. Three points represent age-appropriate function, and 0 points indicate no residual function. A total score is calculated (range 0-15). Within the last year, the participants were assessed with the Norwegian version of the scale^14^ by the third author (I.H.), a child neurologist who has significant experience in clinical assessment of CLN3.

#### Current medications

Data on the medications currently prescribed to participants were obtained from their medical records and from the diagnostic psychiatric interview with parents (discussed below). These included the following psychotropics: stimulants (eg, methylphenidate), selective serotonin reuptake inhibitors (SSRIs), alpha-2 agonists, and antipsychotics. We also included hypnotics because of the significant interrelationship between the development and prognosis of psychiatric disorders and their co-occurring sleep disorders.^15^ Furthermore, we reported on the frequency of medication commonly used by individuals affected by CLN3, including antiepileptics, benzodiazepine derivatives, dopaminergic medications, and other medications (eg, paracetamol, gabapentin). For each medication, the indication for treatment was recorded. Information from the two sources was cross-referenced to ensure accuracy and completeness. Discrepancies, such as medications noted in the diagnostic interview but absent from the medical record, were clarified through follow-up with the prescribing physician or additional parental reporting when possible.

#### Neurodevelopmental and psychiatric disorders and global psychosocial functioning

To assess psychiatric disorders in individual with CLN3, parents were interviewed using the revised version of the semistructured Schedule for Affective Disorders and Schizophrenia for School-Aged Children: Current and Lifetime Version (K-SADS-PL) and DSM-5.^16^ The interview is designed for assessment of individuals up to age 18 years but has also been applied to populations that include young adults.^17^ Satisfactory convergent and discriminative validity and acceptable values for interrater reliability have been reported for the former US and Nordic versions of K-SADS-PL.^18–20^ For the DSM-5 version of K-SADS-PL, a Nordic study found fair to excellent interrater reliability estimates, with most diagnoses in the excellent (κ > 0.75) range.^21^ Excellent interrater reliability (κ ≥ 0.8) was also found in a Japanese study for neurodevelopmental disorders, such as autism spectrum disorder (ASD) and attention-deficit hyperactivity disorder (ADHD).^22^

The last author (K.R.O.), an experienced child and adolescent psychiatrist, conducted the K-SADS-PL diagnostic interviews with parents. The interviews were completed by telephone because of large geographical distances; this form of administration has previously been found to be valid.^23,24^ The interviewer assessed symptoms (including severity and duration) in the current episode, as well as the most severe past episode, to establish current and lifetime diagnoses, respectively. Psychiatric disorders were considered present when the child met *all* DSM-5 criteria for a given disorder, including the required symptom counts, duration, onset requirements, and functional impairment. Isolated symptoms above threshold were also counted when the other symptoms required to meet full diagnostic criteria were absent. Subthreshold symptoms were recognized as present when the child exhibited significant symptoms, but not to the extent (with the intensity, duration, and functional impairment) required to meet diagnostic criteria.

The main diagnostic criteria for the relevant diagnoses are presented in Supplement 1.

The Children's Global Assessment Scale (CGAS) is a clinician-rated tool used to indicate the lowest overall level of the individual's psychosocial functioning (at home, at school, with peers).^25^ Scores range from 0 to 100, with higher scores indicating better functioning. The CGAS is divided into 10-point intervals with a description of an individual's level of functioning for each interval. In a review of the Norwegian CGAS studies, satisfactory evidence was found for convergent, discriminant, and predictive validity, as well as interrater reliability.^26^ A CGAS score of 70 and higher was considered a normal level of psychosocial functioning by the test's original authors.^25^ Empirically derived cutoff points were later found to be 61 for definite pathology.^27^ Although a CGAS of 65 was found in a large sample of children with epilepsy,^28^ the mean CGAS was 52 in a national registry study of children clinically diagnosed with ADHD (n = 11, 119).^29^ The CGAS was assigned by the last author (K.R.O.) directly after conducting the K-SADS-PL interview and considered all information obtained about the affected individual.

#### Ethics

Written informed consent was obtained from participating parents. The Norwegian NCL user association objected to the direct involvement of their offspring with CLN3. The study was approved by the Regional Committee for Medical Research Ethics in Norway (2022/ 379759). Because of the considerable stress associated with receiving a CLN3 diagnosis, permission to seek consent was restricted to those who had been diagnosed more than 1 year prior to the start of the study.

#### Data analysis

The data were analyzed using IBM SPSS Statistics (version 29). We performed descriptive statistics (mean, SD, range) for participant age and CGAS scores, and the number (percentage) of affected individuals with psychiatric diagnoses with each psychiatric diagnoses assessed. Correlation analyses were performed to assess associations between current age with the CGAS and the Hamburg Kohlschütter scale score. The MedCalc for Windows comparison calculator was used to compare 2 independent proportions and difference between means (version 23.1.7; MedCalc Software).

## Results

### Participant Characteristics

A total of 20 individuals (13 males) with CLN3 (80% participation rate) were included at mean age 15.2 years (SD 8.6, range 7-29), with 8 between 7 and 12 years, 7 between 13 and 18 years, and 5 aged ≥19 years. The mean age at onset of CLN3 was 5.7 years (SD 1.2, range 4-8). The mean sum score on the Hamburg Kohlschütter scale was 7.80 (SD 3.78, range 1-14); none had normal vision, see Table 1.

**Table 1.** The CLN3 Symptom Severity Evaluation: The Hamburg Kohlschütter Scale.

Five Areas and Sum	Mean (SD)	Range
Vision	0.60 (0.82)	0–2
Intellect	1.80 (0.77)	0–3
Language	1.70 (0.92)	0–3
Motor function	2.00 (0.92)	0–3
Epilepsy	1.70 (1.22)	0–3
Sum	7.80 (3.78)	0–14

Abbreviation: CLN3, ceroid lipofuscinosis, neuronal 3.

Sixteen of the 20 individuals (80%) lived with their biological parents, whereas the 4 oldest lived in community care. The mean age of the mothers was 47.2 years, and 49.6 years for the fathers (SD = 7.0 years). The parents were well educated, with 50% having completed ≥16 years of education. Because of their offspring's care needs, most parents did not work full-time, the mean percentage of work was 43% for mothers and 72% for fathers, supplemented by a government-funded carer's allowance. Eight of the 20 participants had, at some point, been referred to the Child and Adolescent Mental Health Services.

### Psychiatric Disorders

Among the 20 individuals, 10 fulfilled the criteria for 1 or more current psychiatric diagnoses. An additional 5 met criteria for a past diagnosis, resulting in a lifetime occurrence of psychiatric disorders in 15 individuals (75%). See Table 2 for an overview.

**Table 2.** Past and Current and Total Number of Psychiatric Diagnoses and Subthreshold Present Psychiatric Symptoms in Individuals With CLN3.

Lifetime Psychiatric Diagnoses (DSM-5 codes)	Past	Current	Total	Subthreshold Symptoms
Neurodevelopmental disorders				
Autism spectrum disorder (299.00)	-	1	1	11 (rigidity)
Attention-deficit hyperactivity disorder (314.0)	1	6	7	
Psychotic disorders				6 (hallucinosis)
Depressive disorders				
Depressive episodes (296)	3	-	3	5
Anxiety disorders				
Panic disorder (300.01)	1	-	1	1
Generalized anxiety disorder (300.02)				1
Agoraphobia (300.22)				1
Social anxiety disorder (300.23)				3
Specific phobias (300.29)	-	6	6	2
Adjustment disorder (309)	1	-	1	
Separation anxiety (309.21)	1	2	3	
Obsessive-compulsive disorder (300.3)	1	-	1	3
Elimination disorders				
Enuresis (307.6)				5
Disruptive disorders				
Oppositional defiant disorder (313.81)	1	3	4	5
Any psychiatric disorder^a^	**9**	**18**	**27**	

Abbreviation: CLN3 ceroid lipofuscinosis, neuronal 3.

a Some have several diagnoses, so the number of diagnoses and subthreshold symptoms exceeds the number of individuals in the study.

When grouped, anxiety disorders were the most frequent (n = 11), followed by neurodevelopmental disorders (n = 8), behavioral disorders (n = 4), affective disorders (n = 3), and obsessive-compulsive disorder (n = 1). The most frequent single diagnosis was ADHD (combined or hyperactive-impulsive presentations), accounting for 7 of 8 of the neurodevelopmental disorders, followed by specific phobia (n = 6) within the anxiety disorders category. The specific phobias were as follows: fear of syringes, heights, elevators, flying, furry animals, and spiders (listed in order of frequency).

Current subthreshold psychiatric symptoms were present in all individuals, with behavioral rigidity (inflexible, repetitive patterns, perseveration and resistance to change) as the most frequent (n = 11). Six individuals experienced hallucinations, all of a visual nature. Additionally, 2 of them also had hallucinations related to animals (insects and snakes), 1 auditory and 1 tactile form. Symptoms of enuresis, depression, and oppositional behavior were each found in 5 individuals (see Table 2).

Sex differences were evident, as only 2 of the 13 males (15%) did not meet the diagnostic criteria for psychiatric disorders, compared with 3 of the 7 participating females (43%). However, a χ^2^ test to compare the 2 independent proportions was not significant (χ^2^ = 1.74, *P* = .18).

There were twice as many psychiatric diagnoses for individuals below 18 years (1.28) compared with the adults (0.60), but the difference was not significant.

The mean CGAS score among the 15 individuals who received a diagnosis was 49.56 (SD 10.81, range 35-71). Lower means (indicating a greater impact) were observed in males (46.09, SD 7.57) compared with females (56.5, SD 15.78). However, the comparison between the 2 independent means was not statistically significant (*t* = 1.77, *P* = .10).

There was no significant correlation between CGAS scores and current age or age of symptom onset. The severity of CLN3 (measured by the Hamburg Kohlschütter scale) correlated negatively with age (*r* = –76, *P* < .01), but only a trend was found for less severe CLN3 and an older age of symptom debut (*r* = .35, ns).

We found no significant associations between parental education (measured by years of schooling) and CGAS (as an overall measure of the burden of psychiatric disorders) or number of psychiatric diagnoses. Additionally, there were no significant associations between the Hamburg Kohlschütter scale subscales and CGAS or the number of diagnoses.

**Figure 1.**
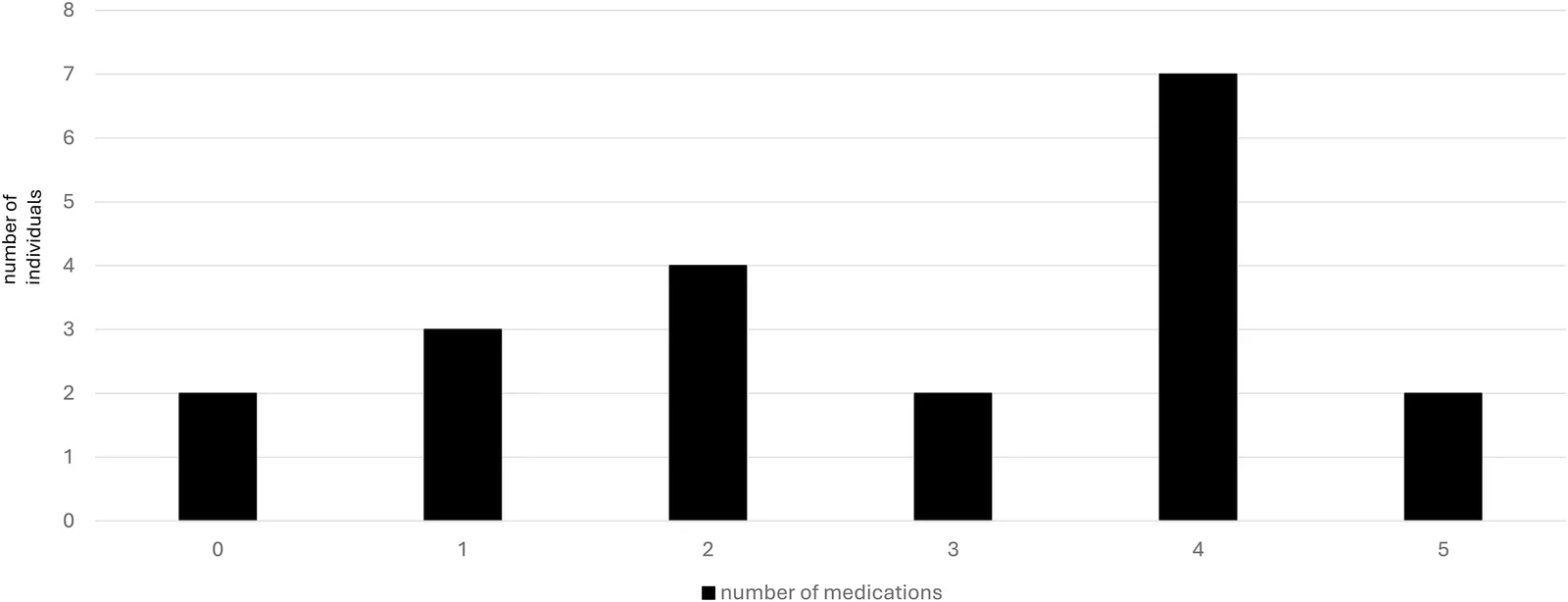
The total number of medications used in participants with CLN3. CLN3, ceroid lipofuscinosis, neuronal 3.

Figure 1 shows that polypharmacy was frequent. Among the 20 individuals, 14 (70%) were on at least 1 antiepileptic medication, with 6 (30%) using more than 1 (2 individuals for <18 years and 4 for >18 years). Additionally, benzodiazepines (eg, diazepam, midazolam) were prescribed to 7 of 20 individuals (35%) for seizure control. Sleep medications were commonly used, with approximately half of the individuals (11/20, 55%) receiving either melatonin and/or alimemazine. Less commonly, antiparkinsonism medications were prescribed to only 2 individuals (10%). Psychotropic medication usage was rare, with only 4 individuals using such treatments, and just 1 receiving 2 psychotropic medications (see Table 3).

**Table 3.** Psychotropic Medications Used, Their Indication, and Number of Individuals on Each Type.

Medications Used	Indication	Number of Participants^a^
*Psychotropics*		
Stimulants: methylphenidate	ADHD	1
SSRIs	Depression, Anxiety disorders, OCD	2
Antipsychotics: risperidone, aripiprazole	Aggression/hallucinations	3
*Hypnotics*		
Melatonin, antihistamines: alimemazin	Sleep	11
*Other*		
Antiepileptics: valproate, lamotrigine, levetiracetam, topiramate	Epilepsy	14
Benzodiazepine derivatives: diazepam, nitrazepam, klonazepam	Acute seizures or status epilepticus	7
Dopaminergic medications: levodopa-carbidopa	Parkinsonism	2
Paracetamol, neurontin	Pain	3

Abbreviations: ADHD, attention-deficit hyperactivity disorder; OCD, obsessive-compulsive disorder; SSRIs, selective serotonin reuptake inhibitors.

a Some individuals use several medications, so the total number of medications prescribed exceeds the total number of participants in the study.

Alpha-2 agonists were not used by our participants.

## Discussion

This study investigated the occurrence of psychiatric disorders in a national sample of individuals with CLN3 and examined the extent to which they received treatment with psychotropic medications. To our knowledge, this is the first study to report clinically registered psychiatric diagnoses and utilize standardized psychiatric interviews for this population.

Our findings indicate that approximately 75% of the sample met the criteria for at least 1 lifetime psychiatric diagnosis, underscoring the significant psychiatric burden in individuals with CLN3. About half of the sample had a current psychiatric disorder, with anxiety and neurodevelopmental disorders (particularly ADHD) being the most common.

Furthermore, all affected individuals displayed subthreshold psychiatric symptoms. This aligns with evidence that subthreshold symptoms of both ADHD^30^ and emotional symptoms^31^ can significantly impact functioning and should not be overlooked.

Similarly, increased risk of psychiatric disorders in individuals with neurologic disorders other than CLN3 is well-documented for more than half a century.^32^ That study reported that psychiatric disorders in children with neuro-epileptic conditions were 5 times as common as in the general population and 3 times as common as in children with chronic physical handicaps not involving the brain. Increased rates were confirmed more recently, for instance, in cohort studies of children with epilepsy^33^ and cerebral palsy.^34^ These risk estimates may stem from both the direct impact of the neurologic disorder (eg, seizures) and the effects of living with a chronic disorder (eg, stress and social stigma).

Consistent with a recent qualitative and quantitative study on CLN3, which reported anxiety symptoms in all 7 of its participants,^6^ anxiety was frequent in our study and was diagnosed in more than half of our sample, with specific phobia being the most common diagnosis. Given that effective treatments exist for these conditions, such as cognitive-behavioral therapy for those with reduced cognitive abilities,^35^ medications,^36^ or a combination of both,^37^ there is significant potential for improving health outcomes. Our previous research demonstrated the effectiveness of adapted cognitive-behavioral therapy for anxiety in children with ASD (entailing short sessions, numerous repetitions of simple and concrete assignments with favorite rewards, close parental involvement during sessions and homework).^38^ Additionally, a case study showed its effectiveness for one child with an anxiety-related obsessive-compulsive disorder and CLN3.^10^ The anxiety disorders identified in the present study differed from the paroxysmal sympathetic hyperactivity episodes with expressions of anxiety and agitation previously described in late adolescence.^39^

ADHD was the most frequent single diagnosis, occurring in 7 of 8 affected individuals with a neurodevelopmental disorder (1 was diagnosed with autism spectrum disorder). Given the cognitive decline associated with CLN3, the high occurrence of ADHD may be controversial. However, our interviews confirmed that ADHD symptoms (hyperactivity, impulsivity, and inattention) were evident in early childhood, preceding the onset of cognitive decline. These symptoms may benefit from targeted and evidence-based interventions, such as psychoeducation, parent management training, pedagogic strategies, and medication.^40^ Less common but notable conditions included oppositional defiant disorder, ASD, and depression. Additionally, hallucinations and rigidity were observed, highlighting the atypical and complex symptom presentations in this population, as previously described in studies using questionnaires.^4,9^ The hallucinations reported by parents in the present study were predominantly visual, which is well-documented in eye diseases and neurodegeneration.^41^ However, in psychosis, these hallucinations often co-occur with auditory hallucinations, inflicting impairment.^42^

In our sample, behavioral rigidity was frequently reported (n = 12) in a manner consistent with that typically associated with ASD. This type of rigidity primarily reflected a reliance on structure and predictability, with insistence on sameness, strict adherence to routines, or difficulties with transitions, as described in the DSM-5.^43^ This finding aligns with a previous study that employed a diagnostic interview on obsessive-compulsive disorder and concluded that participants’ need for routine was not goal-directed or intended to neutralize feared thoughts or events, but instead appeared analogous to perseverative thoughts.^9^

However, we also identified a smaller subset of children (n = 3) in whom rigidity was described as consistent with part of the DSM-5 criteria for obsessive-compulsive disorder. In these cases, the rigidity was part of repetitive, controlled rituals aimed at temporarily alleviating anxiety-driven distress. Differentiating between these forms of rigidity can be challenging, particularly in children who may have difficulty articulating the obsessions or anxieties underlying their behaviours. Nevertheless, making this distinction is important, as the underlying mechanisms require different therapeutic approaches. Of note, obsessive-compulsive symptoms related to perseveration on preferred topics do not involve rituals performed to neutralize anxiety, as is the case with classic obsessive-compulsive disorder.

Males exhibited a higher prevalence of psychiatric disorders than females, with only 2 of 13 males being free of any diagnosis. This aligns with findings that males are more prone to neurodevelopmental disorders, such as ADHD and ASD.^44^ However, sex differences were not significant in our small sample, which is not surprising given the limited number of participants. Still, females also showed substantial rates, with only 3 of 7 not meeting diagnostic criteria, underscoring the need for continued attention to both sexes in future research.

For the psychiatric disorders, the mean CGAS score of 49.56 reflects moderate functional impairment in most social areas or severe impairment in one area.^25^ This indicates significant challenges in daily functioning, being slightly more impaired than children clinically diagnosed with ADHD in the Norwegian Patient Registry (mean CGAS 52.40).^29^

Despite the high occurrence of psychiatric disorders, only 8 of 20 individuals were referred to the local Child and Adolescent Mental Health Services and psychotropic medications were rarely used in the present study. Only 3 individuals in our sample were currently on psychotropic medications: 1 child, 1 adolescent, and 1 adult. This limited usage makes it difficult to draw conclusions about age trends in psychotropic medication use, apart from noting that these were used in all 3 age groups. This underutilization may raise questions about access to psychiatric care in this population. SSRIs and ADHD medications are generally well tolerated and effective in children,^45,46^ but their use may be limited by diagnostic overshadowing,^47^ where the focus on the primary neurologic disorder may obscure comorbid conditions. Also, there may be concerns about side effects from these medications.

Antiepileptic medications were most commonly used and sometimes included benzodiazepines for seizure control. Some of these medications, such as lamotrigine, valproate, and diazepam, may also ameliorate psychiatric symptoms, including mood swings, aggression, and anxiety.^48^ However, the potential side effects of antiepileptics, including psychiatric symptoms, should be further investigated.^49^ Sleep disturbances, a frequent comorbidity, were widely treated with nonaddictive sleep medications (55%), consistent with prior reports of settling problems, nocturnal awakenings, and nightmares in more than half of the individuals.^50^

### Clinical Implications

The high prevalence of psychiatric disorders and the low use of psychotropic medications emphasize the need for multidisciplinary care that integrates psychiatric and neurologic expertise. Routine screening for psychiatric symptoms, including subthreshold symptoms, should be implemented to enable early identification and intervention.

### Limitations

Strengths of the present study include the national sample and the use of a comprehensive diagnostic interview. However, there were several limitations. Because of the rarity of CLN3, the sample size was small. Multisite studies across borders are needed, particularly to confirm the findings regarding sex differences. The partial reliance on retrospective information and incomplete medical records may have affected the accuracy of the data, especially concerning medication use. However, the annual medical assessments follow a stringent procedure and were conducted by an expert on CLN3 (I.H.). The standardized measures employed in the present study (K-SADS-PL and CGAS) were developed for individuals 18 years and younger. Nonetheless, given the cognitive decline in this population, the measures seemed appropriate. Additionally, the K-SADS-PL includes a retrospective component to obtain information about past diagnoses. Although we used the K-SADS-PL, the diagnoses were based solely on parent reports; parents reported behavior across settings (home and kindergarten/school). We did not, however, have information directly from teachers—adding teacher reports to parent reports is considered the gold standard for the clinical evaluation of ADHD.

Although our results suggest underrecognition and treatment of psychiatric disorders among our participants with CLN3, we cannot rule out that they may have received health care for these disorders. All participants will have received care from their primary health care providers, as well as at the pediatric wards of their local hospitals. However, these services do not usually assess or address psychiatric disorders. Local healthcare providers had the option to consult with the child neurologist at Oslo University Hospital for significant difficulties, including for psychiatric disorders. This latter option suggests that we would have been informed if psychiatric disorders were being assessed locally.

Because of the time lag between the neurologist's scoring of CLN3 severity and the psychiatric interviews, we asked the parents to rate the Hamburg scale at the time of the interview and found very high correlations (*r* = .93, *P* = .01). This strengthens the validity of our findings in examining correlations between disease severity and psychopathology.

Longitudinal studies are necessary to follow the progression of psychiatric disorders in individuals affected by CLN3, as these may change over time. The present study could serve as an important foundation for the implementation of such a follow-up-study. Additionally, there is a need for genetic investigations to better understand the psychiatric morbidity associated with CLN3. Such investigations may also be valuable in enhancing our understanding of psychiatric disorders more broadly.

### Summary

This study underscores the substantial burden of psychiatric comorbidity in individuals with CLN3, with significant implications for their overall health and functioning. Integrated care approaches that address the multifaceted needs of these individuals are critical to improving their mental health.

## Supplemental Material

sj-docx-1-jcn-10.1177_08830738251413827 - Supplemental material for Neurodevelopmental and Psychiatric Disorders and the Use of Psychotropic Medications in a National Sample of Individuals With Juvenile Neuronal Ceroid Lipofuscinosis Supplemental material, sj-docx-1-jcn-10.1177_08830738251413827 for Neurodevelopmental and Psychiatric Disorders and the Use of Psychotropic Medications in a National Sample of Individuals With Juvenile Neuronal Ceroid Lipofuscinosis by Beate Oerbeck, Ingrid B. Helland, Heather R. Adams and Kristin Romvig Overgaard in Journal of Child Neurology
