# Supplementary material for: Neurodevelopmental and Psychiatric Disorders and the Use of Psychotropic Medications in a National Sample of Individuals With Juvenile Neuronal Ceroid Lipofuscinosis
Source: J Child Neurol. 2026 Feb 5;41(9):1283–92. doi: 10.1177/08830738251413827 (PMC13522425; doi:10.1177/08830738251413827)
Supplement: Supplementary file 1 [file sj-docx-1-jcn-10.1177_08830738251413827.docx]

**Supplement 1.** The main diagnostic criteria for DSM-5 disorders in the present study.

| DSM-5 diagnoses | Main diagnostic criteria |
| --- | --- |
| 299 ASD | Persistent differences in social communication and interaction, along with restricted, repetitive patterns of behavior, interests, or sensory sensitivities. These symptoms must be present from early childhood, significantly impacting daily functioning |
| 314.0 ADHD | A persistent pattern of inattention and/or hyperactivity-impulsivity that interferes with functioning or development |
| 296.2 Depressive episodes | Period of at least 2 weeks during which there is either depressed mood or the loss of interest or pleasure in nearly all activities |
| 300.01 Panic disorder | Recurrent unexpected panic attacks. A panic attack is an abrupt surge of intense fear or discomfort that reaches a peak within minutes (with minimum 4/13 specified symptoms) |
| 300.2 Specific phobias | Marked fear or anxiety about a specific object or situation (e.g. flying, heights, animals, injections, seeing blood) |
| 309 Adjustment disorder | The development of emotional or behavioral symptoms in response to an identifiable stressor(s) occurring within 3 months of the onset of the stressor(s). With depressed mood: Low mood, tearfulness or feeling of hopelessness |
| 309.21 Separation anxiety | Developmentally inappropriate and excessive fear or anxiety concerning separation from those to whom the individual is attached |
| 300.3 OCD | Presence of obsessions (recurrent and persistent thoughts/urges), compulsions (repetitive behaviors) or both |
| 313.81 ODD | A pattern of angry/irritable mood, argumentative/defiant behavior, or vindictiveness lasting at least 6 months |

***Note:*** ASD Autism Spectrum Disorder; ADHD Attention-deficit hyperactivity disorder; OCD Obsessive-compulsive Disorder; ODD Oppositional Defiant Disorder
